# Baicalein inhibits pancreatic cancer cell proliferation and invasion via suppression of NEDD9 expression and its downstream Akt and ERK signaling pathways

**DOI:** 10.18632/oncotarget.16912

**Published:** 2017-04-07

**Authors:** Rong-Tao Zhou, Mei He, Ze Yu, Yang Liang, Yuzhe Nie, Sheng Tai, Chun-Bo Teng

**Affiliations:** ^1^ College of Life Science, Northeast Forestry University, Harbin, China; ^2^ Department of General Surgery, The Second Hospital of Harbin Medical University, Harbin, China

**Keywords:** baicalein, pancreatic cancer, NEDD9, Akt, ERK

## Abstract

Baicalein, a flavone ingredient of *Scutellaria baicalensis* Georgi, is a promising anti-cancer agent. However, its potential anti-pancreatic cancer effects and the underlying mechanisms are still unclear. In this study, we showed that Baicalein not only induced apoptosis, but also suppressed proliferation, migration and invasion of two pancreatic cancer cell lines BxPC-3 and PANC-1 in a dose- and time-dependent manner. Notably, Baicalein exhibited low toxicity to normal human liver or kidney cells. We further discovered that Baicalein suppressed BxPC-3 and PANC-1 cell proliferation and invasion through targeting the expression of NEDD9, a Cas scaffolding protein, to decrease Akt and ERK activities. Especially, Baicalein decreased Akt phosphorylation at T-308 via lowering NEDD9-dependent PDK1 expression. Overexpression of NEDD9 effectively rescued proliferation and invasion of BxPC-3 and PANC-1 cells dampened by Baicalein. Taken together, our findings suggest that Baicalein is a potent remedy applied to pancreatic cancer treatment in the future.

## INTRODUCTION

Pancreatic cancer, especially the pancreatic ductal adenocarcinoma, is a devastating disease with high mortality and has a 5-year survival rate less than 5%. Its symptom is relatively latent at the early stage rendering a poor prognosis for the suffering [1]. In most cases, once diagnosed, the cancer has already reached the advanced stages characterized by remarkable aggression and malignancy. In fact, distant metastasis accounts for 90% death caused by pancreatic cancer, as revealed by clinical inspections [2, 3]. Accordingly, the effectiveness of surgical resection therapy for pancreatic cancer is greatly reduced. Chemotherapy and radiation therapy are the supplementary schemes; however, pancreatic cancer cells are highly resistant to the traditional chemotherapeutic agents, such as Gemcitabine [4]. To date, there is still lack of potent and low-toxic medication available for the treatment of pancreatic cancer patients.

Nowadays, plant-derived active components attract more and more attention as they are playing extensive roles in treatment of an increasing number of diseases. *Scutellaria baicalensis* Georgi, a traditional Asian herb, is widely used in clearing heat dampness and purging fire detoxification. The main active ingredients of this plant are flavonoid compounds, including Baicalein, Baicalin, Chrysin, Wogonin, and Wogonoside [5, 6]. Among them, Baicalein (5,6,7-trihydroxyflavone) is the most attractive component with a variety of pharmaceutical effects, such as antioxidation, antithrombosis, bacteria- and virus-killing properties, as well as inhibition of inflammatory response and allergic edema [7, 8].

Notably, Baicalein has recently been discovered for its activity against a wide range of cancers, including breast cancer, prostate cancer, ovarian cancer, bladder cancer [9–13]. Baicalein is also found to repress growth and promote apoptosis of several pancreatic cancer cell lines through blocking the 12-lipoxygenase pathway and activating the mitochondrion-dependent apoptotic pathway [14–16]. However, both the detail effects of Baicalein on the pancreatic cancer and the underlying molecular mechanisms are still elusive.

It has been reported that Baicalein can inhibit cancer cell progression through suppressing Akt, MAPKs (ERK/p38), Wnt, and TGF-β signaling pathways [17, 18]. Among them, inhibition of Akt signaling leads to decreased phosphorylation of the downstream mammalian target of rapamycin (mTOR) to arrest cell cycle and induce cell apoptosis or autophagy [19–22], whereas ERK signaling suppression results in downregulation of matrix metalloproteinases (MMPs) but upregulation of the tissue inhibitor of metalloproteinases (TIMPs) to reduce cell motility and migration [23, 24]. Therefore, blocking Akt and/or ERK signaling cascades is an important tactics employed by Baicalein to achieve its anti-tumor activities [25–27]. However, the targets of Baicalein upstream of the Akt and ERK signaling pathways are still understudied.

Neural precursor cell expressed developmentally downregulated 9 (NEDD9), also named as human enhancer of filamentation 1 (HEF1) or Cas-L (Crk-associated substrate L), is a scaffold protein localized in focal adhesions to assemble the focal adhesion kinase (FAK) and the non-receptor tyrosine kinase c-Src to regulate multiple cellular signaling pathways [28, 29]. NEDD9 is highly expressed in breast cancer, colorectal cancer and head and neck cancer, in which its expression levels are positively correlated to cancer cell migration, invasion, and metastasis [30–33]. Moreover, both mRNA and protein levels of NEDD9 are elevated in pancreatic carcinoma compared with the matched adjacent noncancerous tissues [34, 35]. However, there is so far limited information on NEDD9 as a drug target in pancreatic cancer treatment. In this article, we systematically analyzed the effects of Baicalein on pancreatic cancer development, and explored the role of NEDD9 in Baicalein-affected cell signaling pathways.

## RESULTS

### Baicalein inhibits malignancy of pancreatic cancer cells *in vitro*

**Figure 1:**
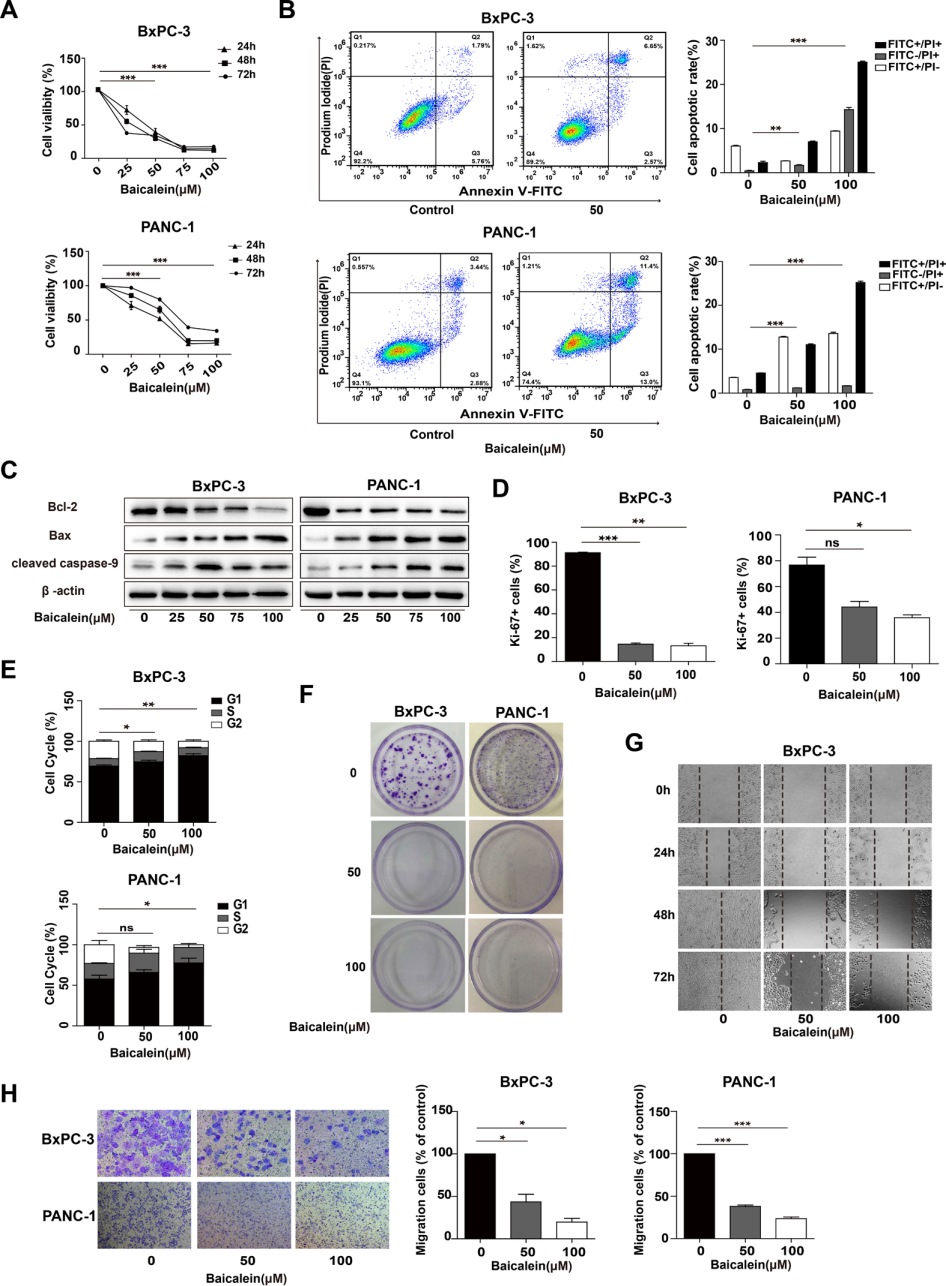
Effect of Baicalein on the viability and motility of pancreatic cancer cells **(A)** Pancreatic cancer cell lines BxPC-3 and PANC-1 were treated with increasing concentrations of Baicalein (25, 50, 75, and 100 μM). DMSO was used as control. Cell proliferation was measured by the CCK8 assay at 24 h, 48 h, and 72 h, respectively. **(B)** BxPC-3 and PANC-1 cells were treated with 50 or 100 μM Baicalein for 48 h, and then subjected to double staining with Annexin V-FITC and PI for flow cytometry detection. The percentages of FITC^+^ and PI^+^ cells were calculated by Flow Jo. **(C)** After BxPC-3 and PANC-1 cells were treated with different concentrations of Baicalein (25, 50, 75, and 100 μM, respectively) for 72 h, the total proteins were extracted to detect the changes in the expression of Bcl-2, Bax and Caspase-9 by western blot, β-actin was used as an inner control. **(D)** After BxPC-3 and PANC-1 cells were treated with 50 or 100 μM Baicalein for 48 h, the proliferating cells were detected by Ki-67 immunofluoresence staining. The bar graphs represent the ratios of Ki-67^+^ cells to total cells. **(E)** Cell cycle was analyzed by flow cytometry after PI staining. The bar graph represents the number of the cells in different phases. **(F)** BxPC-3 and PANC-1 were treated with 50 or 100 μM Baicalein, respectively. After 2 weeks of continuous cultivation, colony forming ability was detected by crystal violet staining. **(G)** BxPC-3 cells were scratched before treatment with 50 or 100 μM Baicalein, and then photographed at 24 h, 48 h and 72 h, respectively. **(H)** Cell invasion ability was analyzed by the Transwell assay. The bar graph represents the number of the cells migrating to the bottom layer. *p < 0.05, **p < 0.01, ***p < 0.001.

To extensively explore the effect of Baicalein on pancreatic cancer growth and invasion, two distinct types of human pancreatic cancer cell lines, BxPC-3 (pancreatic adenocarcinoma cells) and PANC-1 (pancreatic ductal cancer cells), were treated with increasing concentrations of Baicalein (0, 25, 50, 75, and 100 μM) for different time periods (0, 24, 48, and 72 h). CCK8 measurements showed that Baicalein significantly decreased cell numbers of both BxPC-3 and PANC-1 in a dose- and time-dependent manner (Figure 1A). Incubation of 50 μM Baicalein for 48 h markedly induced cell death of BxPC-3 and PANC-1; however, the same concentration of Baicalein exhibited little toxicity to the normal human liver cell line HL-7702 or human embryonic kidney cell line 293T (Supplementary Figure 1A). We then implemented fluorescence activated cell sorter (FACS) analysis via Annexin V-FITC and PI double-staining to investigate apoptosis of BxPC-3 and PANC-1 cells treated with 50 or 100 μM Baicalein for 48 h (Figure 1B). After excluding dead cells, we could still detect 7.01 ± 0.52% and 11.03 ± 0.28% apoptotic cells in BxPC3 and PANC-1 cells, respectively (Figure 1B). Western blotting confirmed that apoptotic promoting proteins Bax and cleaved-caspase 9 were markedly upregulated at 72 h by Baicalein compared with DMSO treatment (Figure 1C).

In addition to cell apoptosis, cell number decrease caused by Baicalein might also be due to cell proliferation inhibition. Thus, Ki-67 staining was employed to examine the effect of the treatment by 50 μM Baicalein for 48 h on the proliferation of the two cell lines. As shown in Figure 1D and Supplementary Figure 1B, there were less Ki-67 positive cells in the Baicalein-treated group (14.43 ± 1.62% of BxPC-3, 44.1 ± 6.09% of PANC-1) than in the control (85.67 ± 1.17% of BxPC-3, 73.8 ± 8.59% of PANC-1). FACS analysis further demonstrated that 50 μM Baicalein treatment led to 74.64 ± 1.73% of BxPC-3 and 59 ± 2.56% of PANC-1 cells arrested at G0/G1 phase (Figure 1E and Supplementary Figure 1C). Western blotting indicated that P21 and P27 were both upregulated in Baicalein-treated BxPC-3 cells (Supplementary Figure 1D). We further tested the effect of Baicalein on the colony-forming ability of BxPC-3 and PANC-1 cells. As shown in Figure 1F, there were only a few colonies observed in 50 μM Baicalein-treated groups and nearly no colony in 100 μM Baicalein-treated groups for both cell lines (n = 3).

The effects of Baicalein on the motility and invasion capability of pancreatic cancer cells were evaluated through wound healing assay and Transwell assay, respectively. At 72 h after BxPC-3 and PANC-1 cells were scratched, the edges of the monolayer cell gaps in the control group had completely merged. In contrast, there were only few cells migrating into the scratched area in the Baicalein-treated group (Figure 1G and Supplementary Figure 1E). In the Transwell assay, based on the number of the cells having migrated to the bottom layer, it could be ascertained that Baicalein significantly inhibited the invasion of both BxPC-3 and PANC-1 cells (Figure 1H). Altogether, the above results showed that Baicalein could not only promote apoptosis, but also suppress proliferation, migration and invasion of the two types of pancreatic cancer cells, thus effectively blocking cancer cell proliferation and invasion *in vitro*.

### Baicalein suppresses Akt and ERK signalings, as well as the expression of NEDD9 in pancreatic cancer cells

**Figure 2:**
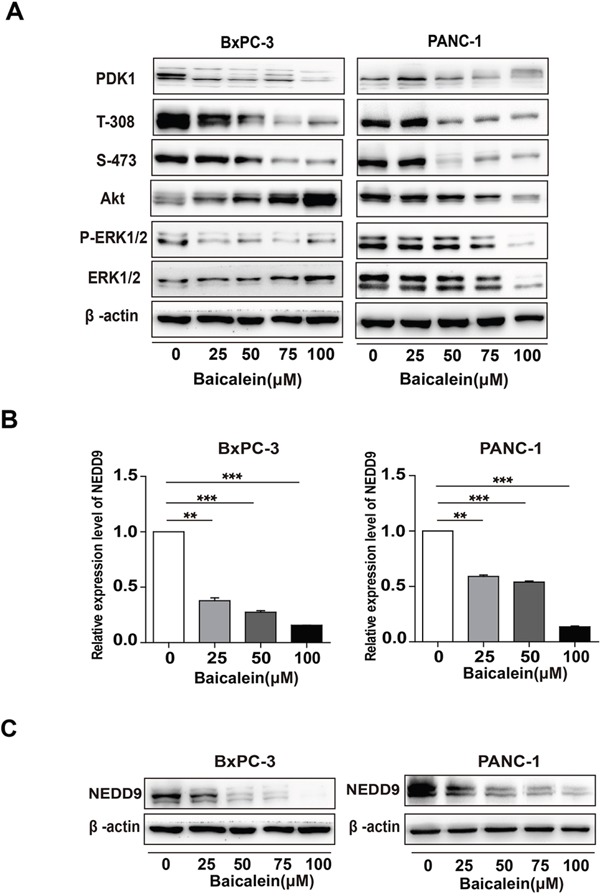
Effect of Baicalein on Akt and ERK1/2 phosphorylation and NEDD9 expression in pancreatic cancer cells **(A)** After BxPC-3 and PANC-1 were treated with different concentrations of Baicalein (25, 50, 75, and 100 μM) for 72 h, the protein levels of PDK1, Akt, and ERK1/2, as well as the phosphorylation levels of Akt on T-308 and S-473, and that of ERK1/2 (p-ERK1/2) were examined. β-actin was used as an inner control. **(B)** BxPC-3 and PANC-1 were treated with different concentrations of Baicalein (25, 50, 75, and 100 μM). DMSO was used as control. After treatment for 48 h, the mRNA level of NEDD9 was detected by qRT-PCR. ** *p* < 0.01, ***p < 0.001. **(C)** After treatment for 72 h, the protein level of NEDD9 was detected by western blot.

As the activation of PI3K/Akt and/or MEK/ERK is important for the invasion of human pancreatic cancer cells [36, 37], we investigated the effects of Baicalein on these two signaling pathways in BxPC-3 and PANC-1 cells. Western blotting results showed that Baicalein indeed decreased the phosphorylation levels of Akt and ERK in a dose dependent manner (Figure 2A), especially the phosphorylation of Akt at Threonine 308 (T-308). Since the T-308 of Akt is mainly phosphorylated by PDK1, we further determined the expression of PDK1. As anticipated, Baicalein also reduced the level of PDK1 in BxPC-3 and PANC-1 cells (Figure 2A). To confirm the roles of PI3K/Akt and MEK/ERK pathways on pancreatic cancer cells, we treated BxPC-3 and PANC-1 cells with PI3K inhibitor LY294002 and MEK inhibitor PD98059, respectively. The CCK8 assay and wound healing assay showed that both inhibitors suppressed not only proliferation but also migration of pancreatic cancer cells (Supplementary Figure 2A and Figure 2B). Compared with PD98059, the inhibitory effects of LY294002 were more pronounced, suggesting that PI3K/Akt activation is more important for pancreatic cancer cell development, which was further supported by the FACS results that LY294002 caused more remarkable apoptosis than PD98059 to BxPC-3 and PANC-1 cells (Supplementary Figure 2C).

Considering that NEDD9, which is involved in multiple Ras-related signalings including Akt and ERK, is highly expressed in pancreatic cancer tissues, we hypothesized that Baicalein may play a role on NEDD9 expression. Thus, we examined NEDD9 mRNA and protein levels in BxPC-3 and PANC-1 cells treated by Baicalein for 48 h and 72h. The results showed that in both cells, Baicalein considerably decreased not only the mRNA level of NEDD9 (Figure 2B), but also its protein level in a dose-dependent manner (Figure 2C).

### NEDD9 knockdown suppresses Akt and ERK signalings, also the expression of PDK1 in pancreatic cancer cells

**Figure 3:**
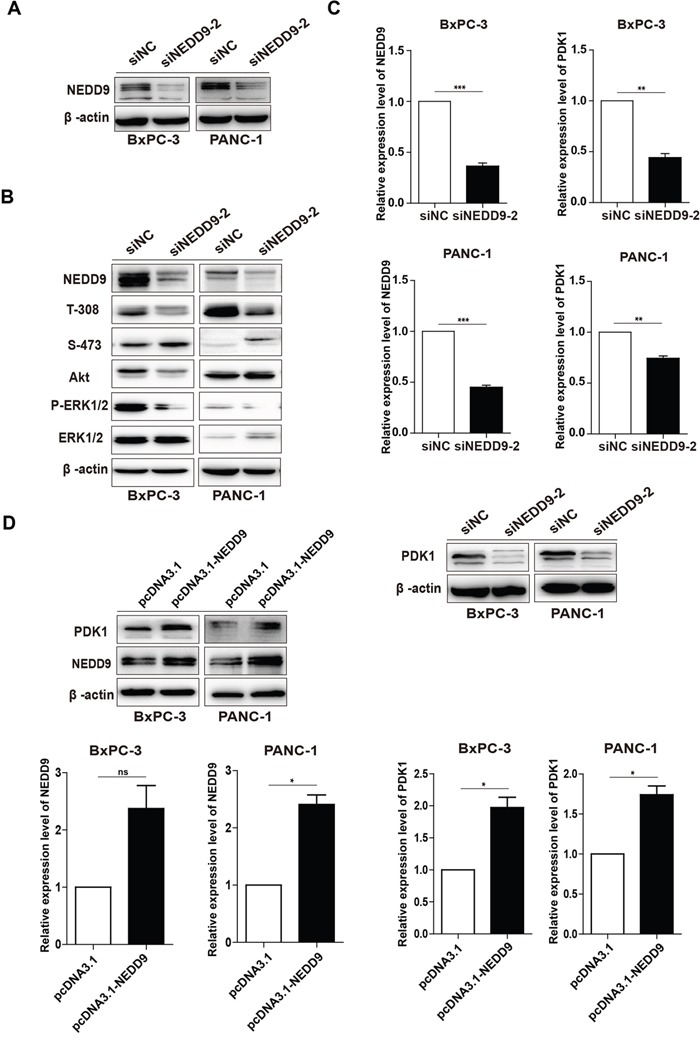
Effects of NEDD9 on phosphorylation of Akt and ERK1/2 and the expression of PDK1 in pancreatic cancer cells **(A)** siNEDD9-2 could efficiently suppress NEDD9 expression in both cell lines. **(B)** After BxPC-3 or PANC-1 cells were transfected with 60 nM siNC or siNEDD9-2 for 72 h, the levels of p-Akt on T-308 and S-473, and p-ERK1/2 were detected. β-actin was used as a loading control. **(C)** After BxPC-3 or PANC-1 cells were transfected with 60 nM siNC or siNEDD9-2 for 72 h, the mRNA and protein levels of PDK1 were analyzed. β-actin was used as a loading control. **p < 0.01, ***p < 0.001. **(D)** After BxPC-3 and PANC-1 cells were transfected with a full-length NEDD9 expression vector, the mRNA levels and proteins levels of PDK1 were analyzed. An empty vector acted as the control. *p < 0.05.

To determine the effects of NEDD9 on Akt and ERK signal pathways in pancreatic cancer cells, we blocked its expression by two synthesized NEDD9 siRNAs. As shown in Figure 3A, siNEDD9-2 efficiently knocked down NEDD9 expression in BxPC-3 and PANC-1 cells. The level of phosphorylated Akt (p-Akt) at T-308, but not at S-473, was decreased upon NEDD9 knockdown. The inhibitory effect was also observed on the phosphorylated ERK (Figure 3B).

Our previous results showed that Baicalein could suppress the expression of both NEDD9 and PDK1 and these two gene products locate upstream p-Akt T-308 [30, 38], whereas their relationship is unknown. The results showed that NEDD9 interference decreased, but NEDD9 overexpression increased PDK1 expression at both mRNA and protein levels (Figure 3C and 3D). These results indicate that, in pancreatic cancer cells, NEDD9 knockdown can inhibit Akt and ERK pathways, and also decrease p-Akt at T-308 through suppressing the expression of PDK1.

### NEDD9 knockdown inhibits pancreatic cancer cell malignancy

**Figure 4:**
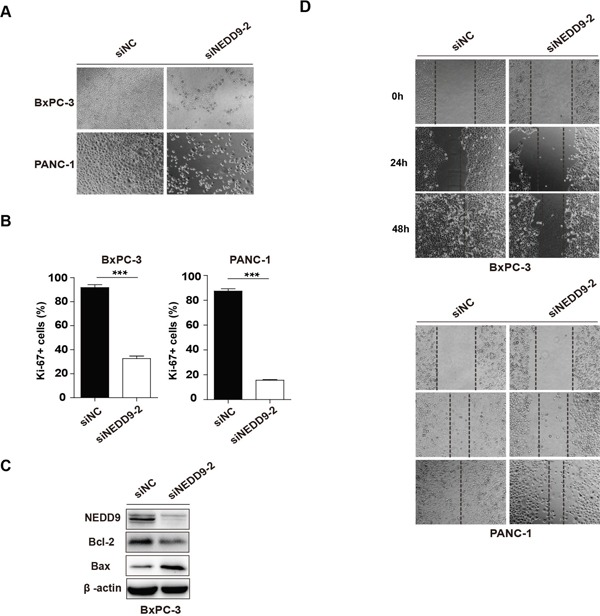
Effects of NEDD9 knockdown on pancreatic cancer cell progression **(A)** 72 h post transfection, the cell morphology was photographed. **(B)** 48 h post transfection, cell proliferation was detected by Ki-67 immunofluoresence staining. The histograms represent the statistical results. ***p < 0.001. **(C)** The apoptosis-related protein Bcl-2 and Bax were examined. **(D)** The migration ability of NEDD9 knockdown cells was analyzed by the wound healing assay at 24 h, 48 h and 72 h, respectively.

Besides, upon NEDD9 interference, we also found that, compared with the negative control siNC, siNEDD9-2 significantly decreased cell viability and increased cell death at 72 h (Figure 4A). Ki-67 immunostaining showed that the proliferation of NEDD9 knockdown cells was obviously suppressed (Figure 4B and Supplementary Figure 3). Western blotting showed that the protein level of Bax was upregulated, while that of Bcl-2 was downregulated after transfection of siNEDD9-2 into BxPC-3 for 72 h (Figure 4C). Moreover, we also carried out wound healing assays to examine the effect of NEDD9 knockdown on cell mobility. As demonstrated in Figure 4D, there were only a few cells that migrated to the scratched area in NEDD9 knockdown groups. These results demonstrated that NEDD9 knockdown induced apoptosis of BxPC-3 and PANC-1 cells as well as decreased their proliferation and mobility, which is consistent with the anti-cancer effects of Baicalein.

### NEDD9 overexpression could rescue the phenotype of Baicalein-treated BxPC-3 and PANC-1 cells

**Figure 5:**
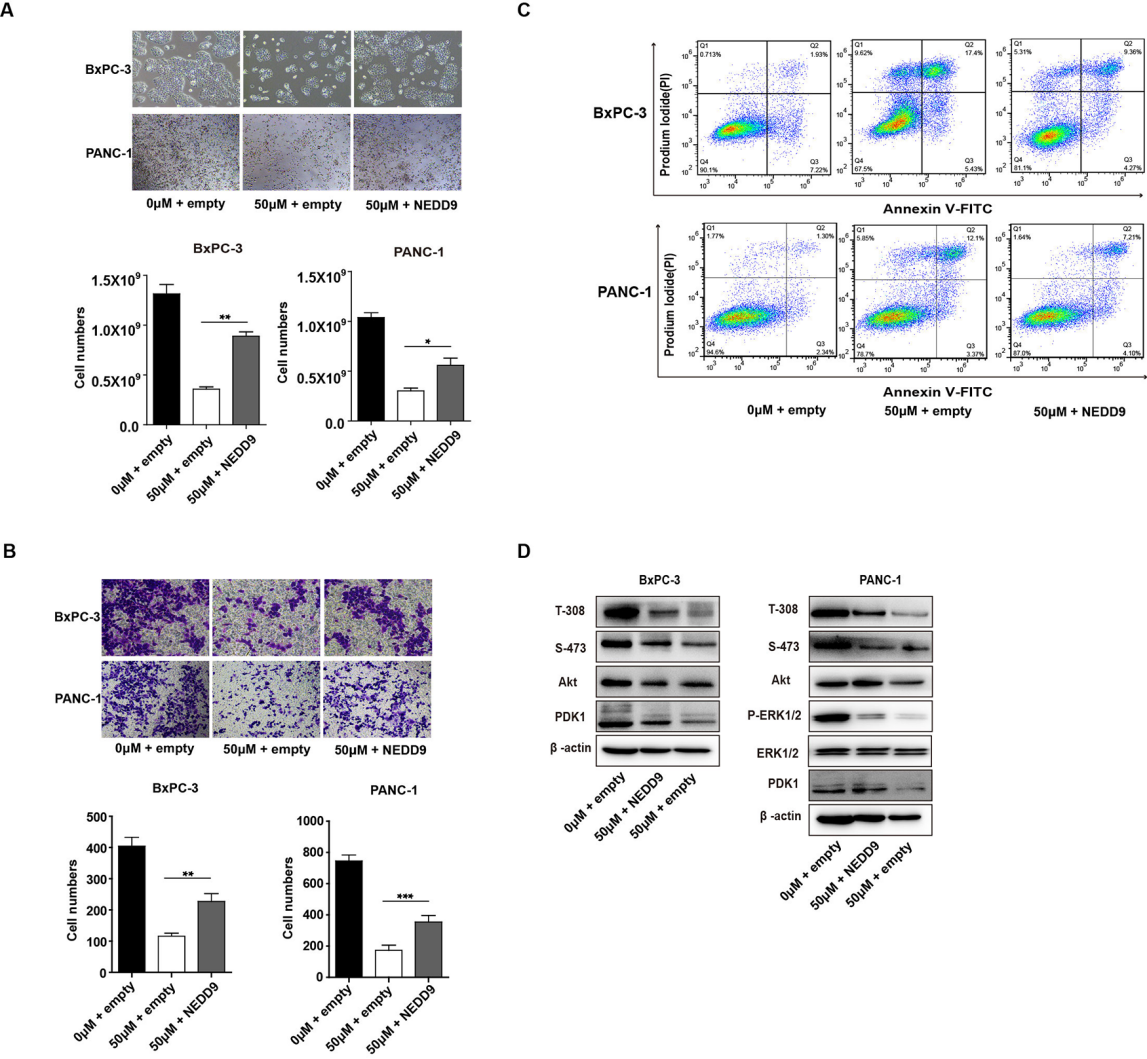
Effect of NEDD9 overexpression on Baicalein-treated pancreatic cancer cell growth and invasion **(A)** After treated with 50 μM Baicalein for 48 h, BxPC-3 and PANC-1 cells were transfected with the expression or empty vector for another 48 h. The viable cells were counted, and the histograms represent the statistical results. **(B)** Cell migration was tested by the Transwell assay and the histograms represent the statistical results. **(C)** Cell apoptosis was studied by flow cytometry analysis of Annexin V-FITC and PI double staining. **(D)** After treated with 50 μM Baicalein for 48 h, BxPC-3 and PANC-1 cells were transfected with the NEDD9 expression vector or empty vector for another 48 h. The protein levels of PDK1, as well as the phosphorylation levels of Akt on T-308 and S-473 and ERK were examined. **p < 0.01, ***p < 0.001.

To explore the role of NEDD9 in the killing property of Baicalein on pancreatic cancer cells, we overepxressed NEDD9 in Baicalein-treated BxPC-3 or PANC-1 cells. The results showed that, with elevated expression of NEDD9, the proliferation of Baicalein-treated BxPC-3 or PANC-1 cells increased (Figure 5A), and the migration and invasion ability gradually enhanced as well (Figure 5B). In contrast, the percentages of apoptotic cells decreased, although the change was not statistically significant (Figure 5C). Moreover, NEDD9 overexpression could rescue Baicalein-decreased Akt phosphorylation at T-308, but not at S-473, and slightly increase ERK phosphorylation (Figure 5D). As expected, NEDD9 overexpression in Baicalein-treated BxPC-3 or PANC-1 cells also restored PDK1 expression (Figure 5D). Taken together, our data revealed that, in the presence of Baicalein, NEDD9 overexpression was able to reactivate Akt and ERK and rescue the phenotype involving proliferation, motility and apoptosis of pancreatic cancer cells, indicating that NEDD9 is a primary target of Baicalein in pancreatic cancer cells.

## DISCUSSION

**Figure 6:**
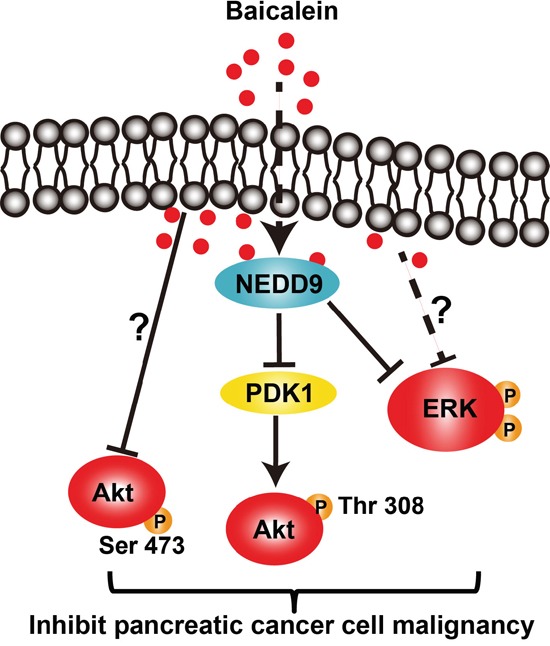
The schematic diagram of the signaling pathways affected by Baicalein in pancreatic cancer cells

In this study, we demonstrated that Baicalein could effectively inhibit the proliferation, migration, and invasion of BxPC-3 and PANC-1 cells, as well as cause a massive cell death, which was achieved through suppressing the PI3K/Akt and MEK/ERK signaling activation in a dose- and time-dependent manner. Further, we discovered that Baicalein could decrease the expression of NEDD9, which was responsible for the inactivation of Akt and ERK by Baicalein treatment. Especially, Baicalein decreased Akt phosphorylation at T-308 via reducing NEDD9-dependent PDK1 expression (Figure 6). Indeed, overexpression of NEDD9 in BxPC-3 and PANC-1 cells treated with Baicalein could effectively rescue their proliferation and invasion.

Baicalein can promote pancreatic cancer cell apoptosis through up-regulating Bax and down-regulating Bcl-2 and Mcl-1, which has been reported by other groups [14, 15]. Here we showed that a defined concentration of Baicalein not only promotes cell apoptosis, but also inhibits the proliferation, colony formation, migration, and invasion of pancreatic cancer cells. Notably, this chemical exhibits low toxicity to normal human cells. Actually, it has already been reported that Baicalein specifically repressed the growth of the cancer cells but not the normal cells, such as normal human fetal lung diploid cell line TIG-1, normal human peripheral blood lymphocytes and normal rat hepatocytes [39–41]. Furthermore, Baicalein administration *in vivo* (20 mg/kg/day p.o.) is able to retard the growth of xenografted prostate cancer cells by 55% as compared with placebo groups after transplantation for 2 weeks [42]; intraperitoneal injection of Baicalein (5, 10, and 20 mg/kg/day; i.p.) can repress orthotopic glioma and human hepatoma models and relieve edema [43, 44]. All these results suggest that Baicalein can be a potent therapeutic medicine on pancreatic cancer.

Our results also showed that Baicalein inhibits pancreatic cancer progression through inactivation of the PI3K/Akt and MEK/ERK signalings, which has been found to be highly active in pancreatic cancers [26]. The inactivation of the Akt and ERK signaling pathways by Baicalein is achieved through downregulation of NEDD9, which is consistent with the previous findings that NEDD9 knockout in a mouse breast cancer model decreases cell proliferation through inhibiting its direct partner FAK and Src, further lowering the Ras-related signaling Akt, ERK and SHCA [45]. However, what is different from the mammary tumor cells is that NEDD9 knockdown in pancreatic cancer cells mainly decrease phosphorlyation of Akt at T-308, but not at S-473. It has been found that AKT phosphorylation at S-473 and T-308 are not always well correlated [46–48], but the reasons are still unknown. Actually, Baicalein inhibits the phosphorylation of Akt at both T-308 and S-473. Our research firstly demonstrated that Baicalein decreased phosphorylation of Akt at T-308 through targeting NEDD9-dependent PDK1 expression, but the target of Baicalein on the suppression of p-AKT at S-473 in pancreatic cancer cells requires further exploration.

It should be pointed out that although Baicalein significantly decreases NEDD9 expression to repress Akt and ERK signalings, NEDD9 might not be a direct target of Baicalein in pancreatic cancer cells. It has been reported that, in breast cancer cells, Baicalein down-regulates Wnt/β-catenin pathway to further inhibit breast cancer cell metastasis [49]. Besides, NEDD9 is demonstrated to be a direct target of Wnt/β-catenin, since there is a TCF binding site on the promoter of NEDD9, which responds to Wnt-3a, β-catenin, and Dvl2 in a dose-dependent manner [31]. Moreover, NEDD9 has been proved by CHIP-Seq to be a downstream target of TGF-β signal pathway, whose activation increases NEDD9 expression and promotes cancer metastasis [50–52]. Molecular docking analysis has demonstrated that Baicalein could specifically bind to the ATP binding site of ALK5 (TGF-β I type receptor) to repress its kinase activity. Thus, Baicalein may also decrease NEDD9 expression through repressing Wnt and/or TGF-β signal pathway and then suppress its downstream Akt and/or ERK activation in pancreatic cancer cells.

In summary, we have identified NEDD9 as a novel target of Baicalein in two different types of pancreatic cancer cells. Through suppressing NEDD9 expression, Baicalein is able to inactivate the Akt and ERK cascades and thus effectively inhibit pancreatic cancer proliferation and invasion. Our findings suggest that Baicalein, with low toxicity to normal human cells, can be a potent remedy applied to pancreatic cancer treatment in the future.

## MATERIALS AND METHODS

### Cell culture and reagents

The human pancreatic cancer cell lines PANC-1, BxPC-3 and 293T were obtained from ATCC, the normal human cell lines HL-7702 cells was generous gifts by Professor GC Sui. The 293T cells were cultured in DMEM Medium with high glucose (Hyclone™) supplemented with 10% fetal bovine serum (FBS) (Biological Industries, 04-001-1A), 1% glutamine (Hyclone™) and 1% penicillin and streptomycin (Hyclone™). All other cells were grown in RPMI-1640 Medium (Hyclone™). Baicalein and inhibitors (LY2940042 and PD98059) were purchased from Sigma and dissolved in DMSO.

### Real-time qPCR

The total RNA was extracted by Tripure™ reagent (Roche, USA) according to the manufacturer's protocol. Each sample was reversed to first strand cDNA using the All-in-One First-Stand Synthesis MasterMix (5X, with DNase I) (NOVA, Yugong Biolabs Inc, Jiangsu, China). All primers used were provided in Supplementary Table 1. The Faststart^®^ Universal SYBR Green Master mix (Roche, Indianapolis, IN, USA) was used in real-time PCR carried out in the LightCycler^®^ 480II Real-Time PCR System (Roche, Basel, Switzerland). Quantification was performed using the second derivative maximum method. mRNA expression levels were normalized to that of β-actin.

### Western blotting

BxPC-3 and PANC-1 cells treated with Baicalein, inhibitors, or siRNAs were lysed in the RIPA buffer (Beyotime Biotechnology). The concentrations of the total proteins were measured using the Bicinchoninic acid (BCA) method (Beyotime Biotechnology). Protein samples (20 μg) were subjected to SDS-PAGE and transferred to nitrocellulose membranes by electroblotting. The membranes were incubated with the primary antibodies and then the corresponding secondary antibodies (horseradish peroxidase-conjugated goat anti-Rabbit IgG or goat anti-mouse IgG at 1:1000 dilution, Beyotime Biotechnology). The signals were detected using Super ECL Detection Reagent (High-sig ECL Western Blotting Substrate, 180-5001, Tanon, Shanghai, China). Primary antibodies used: NEDD9 (#4044, 1:500), Akt (#4691, 1:1000), p-Akt S-473 (#4060, 1:1000), p-Akt T-308 (#2965, 1:1000), ERK1/2 (#9102, 1:1000), p-ERK1/2 (#9101, 1:1000), PDK1(#3062, 1:1000), P21(#8831, 1:1000), P27(#3686, 1:1000) and cleaved caspase-9 (#7237, 1:1000) from Cell Signaling Technology Inc. (Danvers, MA, USA), Bax (AB026, 1:1000) and Bcl-2 (AB112, 1:1000) from Beyotime Biotechnology (Shanghai, China).

### Transient transfection

The pancreatic cancer cells were respectively transfected with 60 nM siNEDD9s and negative control siNC, using Lipofectamine^®^ RNAiMAX (Thermo Fisher Scientific), while pcDNA3.1 plasmids with or without full-length NEDD9 were transfected using Lipofectamine^®^ 2000 according to the manufacturer's instructions (Invitrogen, USA). siRNAs(GenePharma biological company) or plasmid sequences are available in Supplementary Table 2. The RNAi efficiency or gene overexpression was determined by Western blotting.

### CCK8 assay and cell counting

To analyze cell viability, BxPC-3 and PANC-1 cells were seeded at a concentration of 8×10^3^ per 96-well. On the next day, cells were supplied with a fresh medium containing Baicalein, inhibitors, or DMSO (control) or transfected with siRNAs. After 24 h, 48 h, and 72 h of cultivation, 10 μl of the CCK8 reaction solution (Dojindo Laboratories, Japan) was added to each well. After 2-3 h incubation at 37°C in the 5% CO_2_ incubator, the absorbance at 450 nm was measured by a microplate reader (Sunrise™, Tecan, Männedorf, Switzerland). Moreover, BxPC-3 and PANC-1 cells were transfected with the full-length NEDD9 expression vector. After treated with 50 μM Baicalein for 48 h, cell numbers were counted using an automated cell counter (LUNA™ Automated Cell Counter, Logos Biosystems, Korea).

### Colony formation assay

BxPC-3 and PANC-1 cells were seeded in 60 mm dishes at a concentration of 1,000 cells per plate. The cells were treated with DMSO or Baicalein (50 or 100 μM), and then cultured for two weeks at 37°C in the 5% CO_2_ incubator. All colonies were fixed in 4% PFA for 20 min, dried in air, and stained with 0.05% crystal violet.

### Cell-cycle analysis

After BxPC-3 and PANC-1 cells were treated for 48 h, the cells were collected and fixed with ice-cold 70% ethanol for 2 h at 4°C. After centrifugation at 900 rpm for 5 min, the cells were washed and re-suspended in PBS containing propidium iodide (PI; 50 mg/ml) and RNase-A (50 μg/ml). Samples were then incubated at room temperature for 30 min and analyzed by a flow cytometer (BD Accuri™ C6 Plus, BD Biosciences, New York, USA). Cell-cycle phases were analyzed using Modfit™ Software.

### Apoptosis analysis by Annexin V- PI double staining

Apoptosis was analyzed using the Annexin V-PI apoptosis detection kit (A211, Vazyme Biotech Co., Ltd, Nanjing, China). Briefly, the treated BxPC-3 and PANC-1 cells were trypsinized and washed twice with ice-cold PBS and re-suspended in the binding buffer at a concentration of 10^5^ per well in a total volume of 100μl. Then, the cells were supplied with 5 μl of Annexin V-FITC and 5 μl of PI and incubated in dark at room temperature for 10 min. After that, 200 μl of binding buffer was added into each sample for flow cytometry analysis.

### Wound healing assay

BxPC-3 and PANC-1 cells were trypsinized and seeded in a 24-well plate at a concentration of 8×10^4^ cells per well. After overnight incubation, the cell monolayers were scratched using a plastic tip across the plate. The wells were washed three times with PBS to make sure that no suspending cells existed in the wound areas. Subsequently, the cells were incubated in low-serum (2%) medium with treatments and the wound images were taken as 0 h. After 24 h, 48 h, and 72 h, wound healing pictures were taken.

### Cell invasion assay

Cell invasion was tested using a transwell matrigel assay system (3422, Corning Incorporated, USA). The treated cells were seeded with serum-free DMEM medium into the upper layer polycarbonate membrane filter and 20% FBS was added to the bottom chambers. After 48 h or 72 h, the cells on the upper layer were removed with a cotton swab, while the cells on the bottom of the filter were fixed with 4% PFA, stained with 0.05% crystal violet and counted.

### Immunofluorescence staining

The cells were fixed in 4% PFA for 20 min, washed with PBS and permeabilized using 0.3% triton X-100 for 25 min at room temperature. Samples were blocked with 10% normal animal serum for 1 h and incubated with the primary antibody rabbit anti-Ki-67 (NCL-Ki67P, 1:200, Novocastra Laboratories Ltd., Newcastle upon Tyne, UK) for 1 h. Then, cells were washed and incubated for 1 h with the secondary antibody Alexafluor 488 Goat Anti-Rabbit IgG(H+L) (A0423) or Alexafluor 555 Donkey Anti-Rabbit IgG(H+L) (A0453) from Beyotime (Shanghai, China) diluted at 1:1000. The nuclei were stained with Hoechst 33258 (C1017, Beyotime, China) and visualized using a Nikon invert fluorescent microscope. Images were analyzed using Image Pro Plus software.

### Statistical analysis

Each experiment was repeated three times, and significance was determined with two-tailed Student's t-test or two-way ANOVA. Error bars represent ± SEM. *p*-value lower than 0.05 was considered as significant. *p < 0.05, **p < 0.01, and ***p < 0.001.

## SUPPLEMENTARY MATERIALS FIGURES AND TABLES


