# Supplementary material for: Baicalein inhibits pancreatic cancer cell proliferation and invasion via suppression of NEDD9 expression and its downstream Akt and ERK signaling pathways
Source: Oncotarget. 2017 Apr 7;8(34):56351–63. doi: 10.18632/oncotarget.16912 (PMC5593566; doi:10.18632/oncotarget.16912)
Supplement: Supplementary file 1 [file oncotarget-08-56351-s001.pdf]

Baicalein inhibits pancreatic cancer cell proliferation and invasion via suppression of NEDD9 expression and its downstream Akt and ERK signaling pathways

SUPPLEMENTARY FIGURES AND TABLES

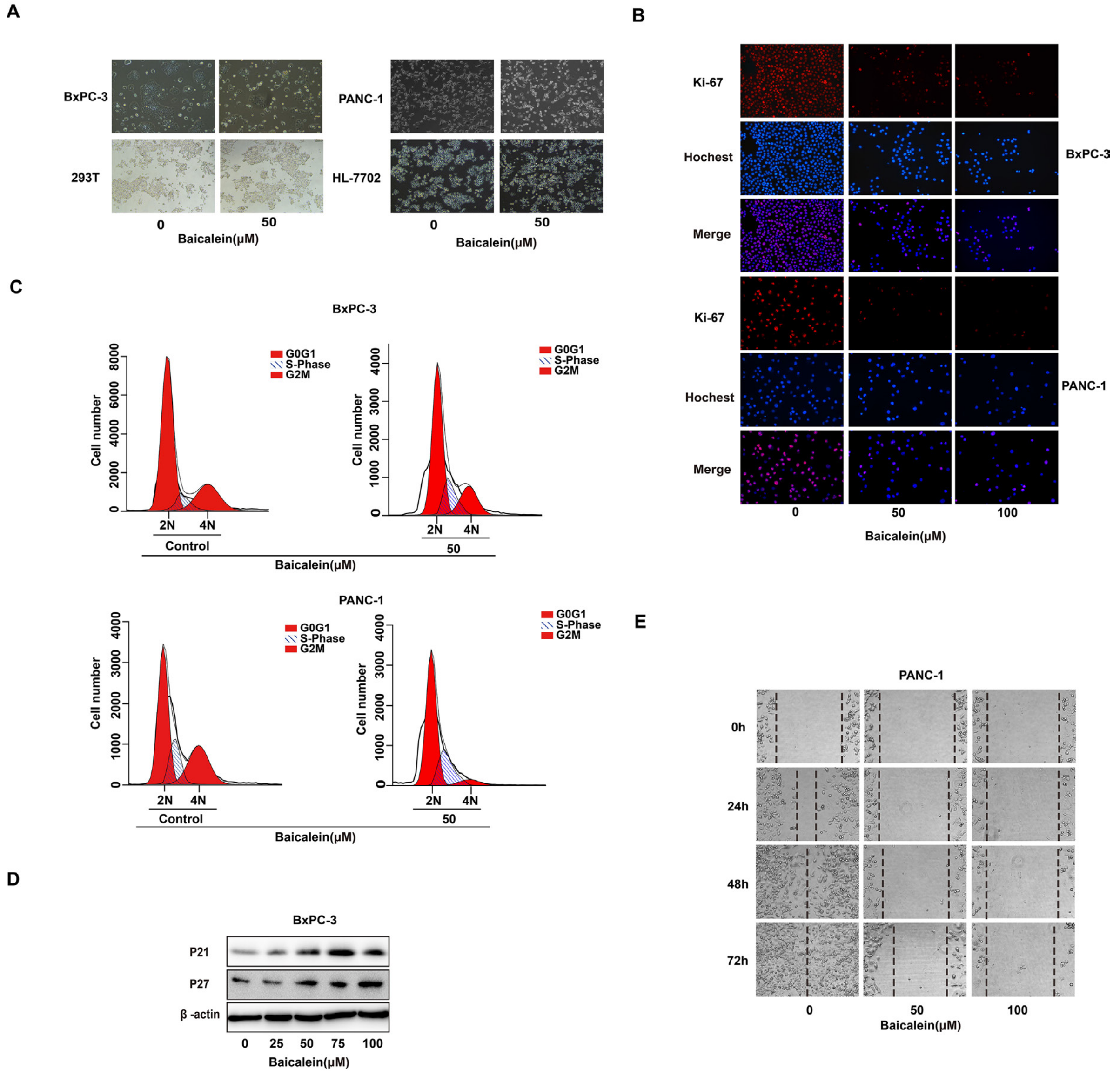

Supplementary Figure 1: Effect of Baicalein on the proliferation, apoptosis, cell cycle and motility of pancreatic cancer cells. (A) The cell morphologies were observed and photographed after BxPC-3, PANC-1, 293T and HL7702 cells were treated with 50 μM Baicalein for 48 h. **(B)** After BxPC-3 and PANC-1 cells were treated with 50 μM Baicalein for 48 h, cell proliferation was presented by Ki-67 immunofluorescent staining. **(C)** BxPC-3 and PANC-1 were treated with 50 μM Baicalein for 48 h, the changes in cell cycle were detected by PI staining. **(D)** After BxPC-3 and PANC-1 cells were treated with Baicalein for 72 h, the protein levels of P21 and P27 were detected. **(E)** The migration ability of PANC-1 cells treated with 50 or 100 μM Baicalein was examined by the wound healing assay at 24 h, 48 h and 72 h, respectively.

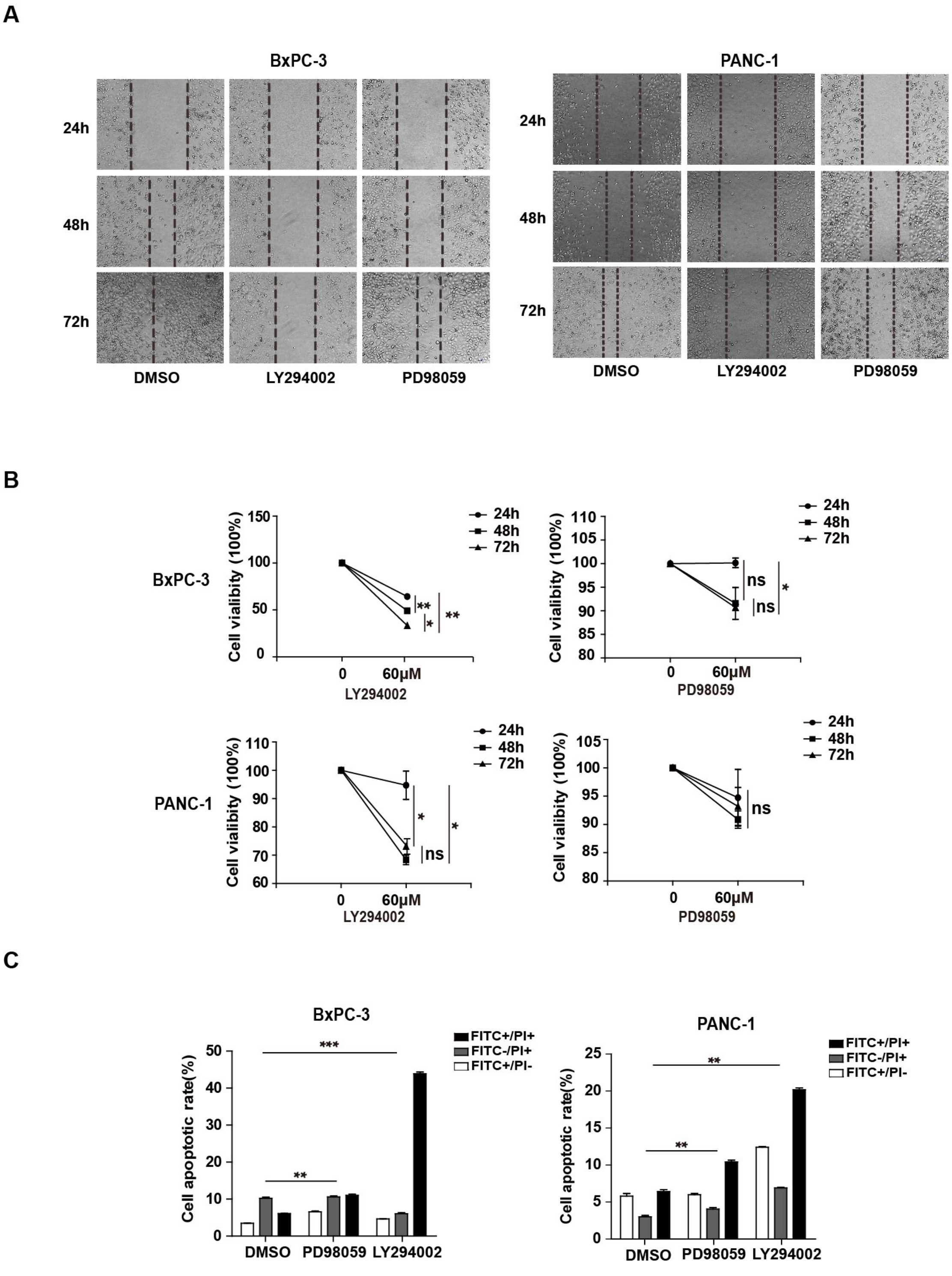

Supplementary Figure 2: Effect of LY294002 and PD98059 on pancreatic cancer cell proliferation, apoptosis and mobility. (A) BxPC3 and PANC-1 cells were treated with 60 μM LY294002 or PD98059. Equal volume of DMSO was used as control. The migration ability of BxPC-3 and PANC-1 was examined by the wound healing assay at 24 h, 48 h and 72 h, respectively. **(B)** The proliferation of BxPC-3 and PANC-1 cells treated with LY294002 or PD98059 was analyzed by the CCK8 assay at 24 h, 48 h and 72 h, respectively. The line graphs represent statistical results. **(C)** BxPC-3 and PANC-1 cells were treated with LY294002 or PD98059 for 48 h, and then subjected to double staining with Annexin V-FITC and PI for apoptosis analysis. The number of the cells in different apoptotic stages was denoted by quantitative graph. $^{*}p < 0.05$, $^{**}p < 0.01$, $^{***}p < 0.001$.

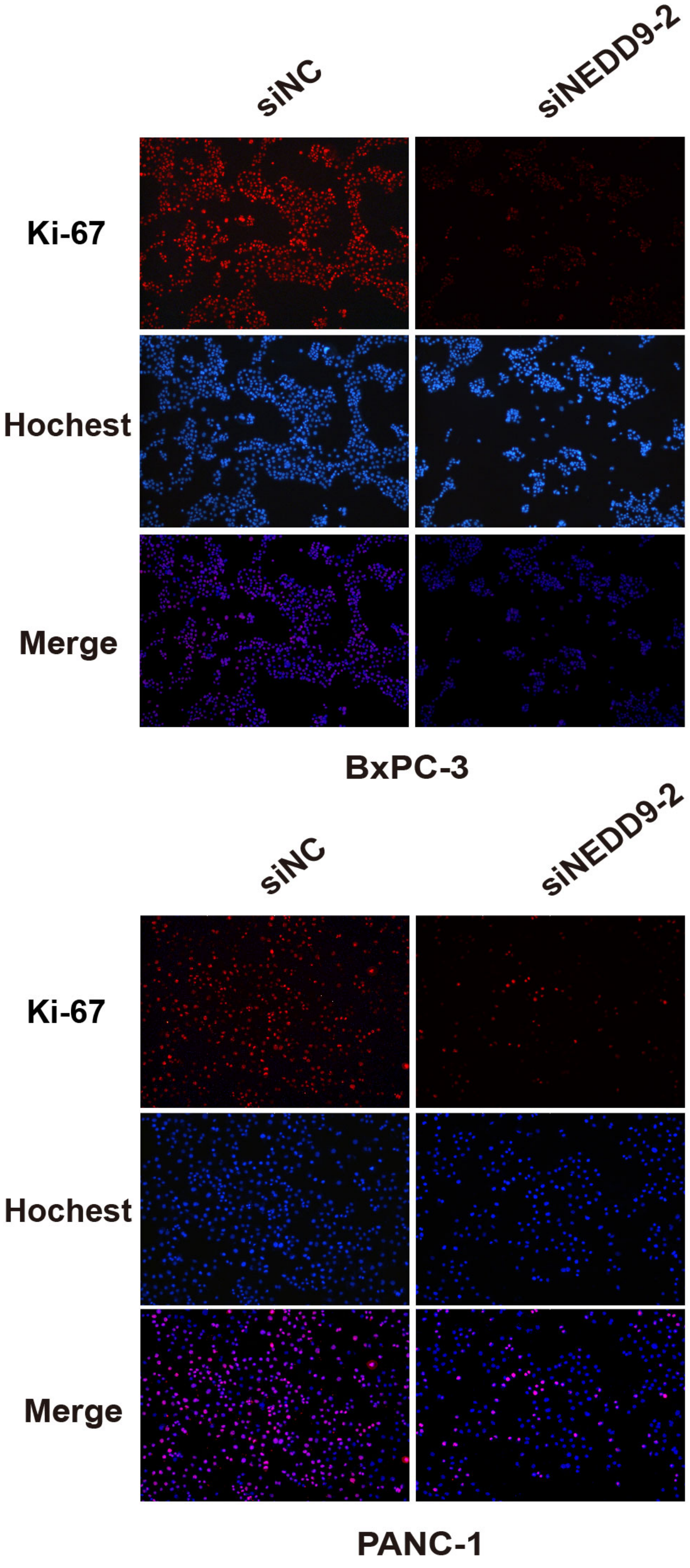

Supplementary Figure 3: Effect of NEDD9 knockdown on the proliferation of pancreatic cancer cells. The proliferation of BxPC-3 and PANC-1 cells upon NEDD9 knocked-down was represented by Ki-67^{+} cells.

Supplementary Table 1: The primer sequences of human NEDD9 and β-actin for qRT-PCR assay

Primer names	Sequence
human NEDD9	Sense 5’-CTGAGCGGAGTTGGATGGATGA-3'
	Antisense 5’-CGTCGATGGCGTTGAGTAGGGA-3'
human β-actin	Sense 5’-CCTGGCGTGGGTAGATTGCTGT-3'
	Antisense 5’-ACGTGGGAAGAGGTGAAGATAAGG-3'

Supplementary Table 2: The siRNA sequences for NEDD9 knock-down

siRNA names	Sequence
siNEDD9-2	Sense 5’-GAGACACCAUCUACCAAGU-3'
	Antisense 5’-ACUUGGUAGAUGGUGUCU C-3'
siNEDD9-1	Sense 5’-CCCACCAGAUUCUAAGCCAAA-3’
	Antisense 5’-UUUGGCUUAGAAUCUGGUGGG-3’
